# Understanding the Link Between Adult Asthma and Coronary Artery Disease: A Narrative Review

**DOI:** 10.7759/cureus.43621

**Published:** 2023-08-17

**Authors:** Vasudha S Garg, Mihir H Sojitra, Tyagi J Ubhadiya, Nidhi Dubey, Karan Shah, Siddharth Kamal Gandhi, Priyansh Patel

**Affiliations:** 1 Department of Internal Medicine, Civil Hospital Ahmedabad, Ahmedabad, IND; 2 Department of Neurology, Civil Hospital Ahmedabad, Ahmedabad, IND; 3 Department of Internal Medicine, Meghji Pethraj (MP) Shah Government Medical College, Jamnagar, IND; 4 Department of Internal Medicine, Medical College Baroda, Vadodara, IND

**Keywords:** adult onset, atherosclerosis, chronic inflammation, coronary artery disease, asthma

## Abstract

Asthma is a common pathology worldwide that occurs due to chronic inflammation of the respiratory airways. Persistent pulmonary inflammation leads to low-grade systemic inflammation, influencing blood vessels and triggering coronary artery disease (CAD) events. This review's objectives include discussing the susceptible population for CAD, the mechanism underlying CAD creation in asthma patients, the characteristics of asthma, and the influence of anti-asthmatic medications on CAD development. Adult-onset asthma is strongly linked to CAD and stroke. Future research may shed light on these disparities. Atherosclerosis and asthma are linked through both intrinsic and extrinsic pathways, with inflammation being the intrinsic pathway and hypoxia and tachyarrhythmia being the extrinsic pathways. The most probable mechanisms for increased coronary vasospastic angina (CVsA) incidence in asthmatic patients are vascular smooth muscle cell hypercontraction and endothelial dysfunction. Studies have shown a dose-response relationship between asthma control and myocardial infarction (MI) risk, with uncontrolled asthma at the highest risk. Impairment of ventilatory function is a distinct risk factor for lethal MI and cardiovascular death (CVD). The use of beta-2-agonists and chronic oral glucocorticoid therapy in severe asthmatics has been linked to increasing the risk for CAD. However, some studies have shown that the risk of MI among patients with active asthma is not related to the use of asthma medications. Further research is needed to determine the involvement of adult asthma features and their treatments in the development of CAD.

## Introduction and background

Asthma is now more widespread than ever among young adults in Western society [1-5], where it is one of the most prevalent chronic disorders. Asthma can result in a person's quality of life being lower and society's healthcare costs rising [2]. An increased risk of death from all causes was correlated with having asthma episodes and nocturnal symptoms [2]. Asthma sufferers should receive good treatment and control in order to reduce the high mortality rate of one death per 100,000 people that exists in developed countries. Asthma, a common pathology, affects about 15%-20% of people in developed countries and approximately 2%-4% in less developed countries [1]. It is a variable chronic inflammatory disease of the airways that results in fluctuating airway hyperresponsiveness and intermittent airflow restriction [2].

Coronary artery disease (CAD) is a leading cause of death worldwide, and despite lowering age-specific mortality rates in affluent countries, an aging population keeps the absolute numbers rising [3]. It has the following subtypes: stable ischemic CAD and acute coronary syndrome (ACS). ACS is further divided into the following types: ST-elevation myocardial infarction (STEMI), non-ST-elevation myocardial infarction (NSTEMI), and unstable angina [4]. It is known that inflammation plays a key role in the pathogenesis of atheromatous thrombus formation, as the major four markers of inflammation, high-sensitivity C-reactive protein (hs-CRP), serum amyloid A, interleukin-6 (IL-6), and soluble intracellular adhesion molecule (sICAM-1), are considered good predictors of the risk of future cardiovascular events [5]. Increased blood levels of CRP and fibrinogen, two markers of systemic inflammation, are linked to airway constriction [6]. Persistent pulmonary inflammation promotes the release of pro-inflammatory mediators and cytokines into the circulation, and the mediators stimulate the production and release of acute-phase proteins and inflammatory cells, resulting in a state of low-grade systemic inflammation, according to a study [6]. Systemic inflammation also has an impact on blood vessels, encouraging the instability and rupture of atherosclerotic plaque and precipitating sudden cardiac and cerebral events [6]. This theory might hold true, as patients with a current status of asthma are associated with a 41% increased risk of cardiovascular disease mortality [7]. The link between these two chronic inflammatory conditions, which contribute to a large share of morbidity and mortality, is still unknown. In our literature review, we delve deeper into the association between adult asthma and its effect on developing CAD and address the susceptible population for CAD, the pathophysiology underpinning the formation of CAD in asthma patients, the characteristics of asthma, and the impact of anti-asthmatic drugs on the development of CAD.

Methodology

In conjunction with all authors, PubMed Central, Medline, and PubMed databases were searched using combinations of asthma and coronary artery diseases. A total of 598 studies were identified, and a free full-text filter was applied to yield 275 papers. Also, the following search strategy was selected based on the Medical Subject Headings (MeSH) vocabulary: ("Asthma"[Majr] AND (alladult[Filter] OR adult[Filter] OR middleagedaged[Filter] OR middleaged[Filter] OR youngadult[Filter])) AND ("Heart Diseases"[Majr]). No time parameters were set. Age criteria of 19-64 years were selected. We selected all types of study literature that was published in English with full text. Articles were excluded where the free full text could not be retrieved. Duplicate publications and gray literature were also excluded. All articles were reviewed, and any differences of opinion were discussed until a decision was reached. After discussion among all the authors, a total of 25 studies were unanimously included in the review.

## Review

Understanding asthma and its subtypes

Asthma is a chronic inflammatory disorder of the lung airway system that clinically presents as shortness of breath (SOB), wheezing, coughing, and chest tightness due to the interplay of inflammatory cells [8]. The T-helper 2 (Th2) cells of the immune system secrete a group of cytokines, including IL-4, IL-5, IL-9, and IL-13. B cells are activated by IL-4, which releases immunoglobulin E (IgE), which is captured by mast cells. Cross-linking of such immunoglobulins on mast cells results in degranulation and the subsequent release of toxic substances, such as leukotrienes (LTs). They, in turn, enhance glycoprotein production, promote eosinophilia, and cause airway hyperreactivity. IL-13 also elicits airway hyperreactivity by acting on the smooth muscles and epithelium of the lung airways [8]. The past few decades have witnessed an overall rise in the prevalence of asthma, with a significant rise affecting the adult population. Earlier distinguished as "extrinsic" and "intrinsic" asthma, the classification of asthma has now been shifted based on the age of onset: childhood-onset (or allergic) asthma and adult-onset (or non-allergic) asthma. When asthma symptoms present for the first time during adulthood, they are classified as adult-onset asthma [9]. Adult-onset asthma is different from childhood asthma due to its poor control, non-atopic features, and rapid decline in lung function [10].

Understanding CAD

CAD presents as an occlusion of the coronary arteries, the major vessels supplying the heart. This, in turn, may lead to myocardial infarction (MI), ischemia, and death. The pathophysiology behind this condition involves the formation of atheromatous plaques within the intima of the vessel wall, along with severe inflammation of the same. This causes a reduced supply of oxygen and nutrients to the cardiomyocytes [11]. It has the following subtypes: stable ischemic CAD and ACS. The latter is further divided into the following types: STEMI, unstable angina, and NSTEMI [4]. In addition to conventional atherosclerotic CAD, coronary vasospastic angina (CVsA) plays an important role in MI, including stable angina, ACS, and fatal cardiac events [12]. The pathophysiology of CVsA is different from those of conventional atherosclerotic angina. Endothelial dysfunction can result from either adventitia or endothelium-related inflammation. The increased rho-kinase is then used by the endothelial dysfunction to further cause vascular smooth muscle hypercontraction, leading to the clinical event [12].

Epidemiology of CAD in asthmatics

The age of onset and current age of presentation of asthma play a huge role in the development of CAD. According to a prospective study that examined the etiology of atherosclerosis, cardiovascular disease, and cerebrovascular disease in four US communities, adult-onset asthma, rather than child-onset asthma, was found to be strongly linked with the incidence of CAD and stroke. This study classified asthma as "adult-onset asthma" if the age of onset was 21 years or older or "child-onset asthma" if the onset was before age 21 [13]. Another study has mentioned that CAD and financial barriers to asthma-related healthcare were substantially associated with asthma-related hospitalizations in older persons with asthma [14]. Another striking epidemiological property of asthma-associated CAD patients was the variation with gender. It was reported that women with adult-onset asthma had a twofold increase in the rate of CAD compared to their non-asthmatic counterparts, which was irrespective of other risk variables such as smoking, body mass index (BMI), and physical activity, and results were congruent when the study was confined to non-smokers [13]. This was backed up by another retrospective cohort study, which demonstrated that asthma increases CAD risk in females by 1.24-fold [15]. Researchers have attributed these disparities mainly to the role of female sex hormones [16]. Contrary to this, males with active asthma had a 63% increased risk of developing CAD than non-asthmatics [17].

Pathophysiology

Researchers and scholars have put forward many hypotheses to explain the association of CAD with adult asthma. Asthma may predispose to atherosclerosis through certain pathophysiologic routes that may be linked to the long-term inflammatory process of this disorder. Alternatively, the link between atherosclerosis and asthma may be caused by a common inflammatory pathway that predisposes people to both conditions [13]. The immune system has two arms: the type one response, which generates ILs, tumor necrosis factors (TNF), granulocyte-macrophage colony-stimulating factor (GM-CSF), and interferons (IFNs), and the type two response, which secretes ILs and encourages allergic inflammation. There is evidence that patients with asthma have higher TNF-alpha levels, even though asthma is primarily a Th2 condition [15]. TNF-alpha and IL-6, two pro-inflammatory cytokines associated with higher levels in asthma, are crucial in the etiology of atherosclerosis. The human atherosclerotic plaque has been reported to include IL-6, which is a key inducer of acute-phase reactants such as CRP. IL-6 can also stimulate platelets and smooth muscle cell mitogenic activity [15]. TNF-alpha, which was first identified by its antitumor effect, has also been found in human atherosclerotic plaques and has been linked to insulin resistance, fibrinogen regulation, factor VIII regulation, and fibrinogen modulation [15].

**Figure 1:**
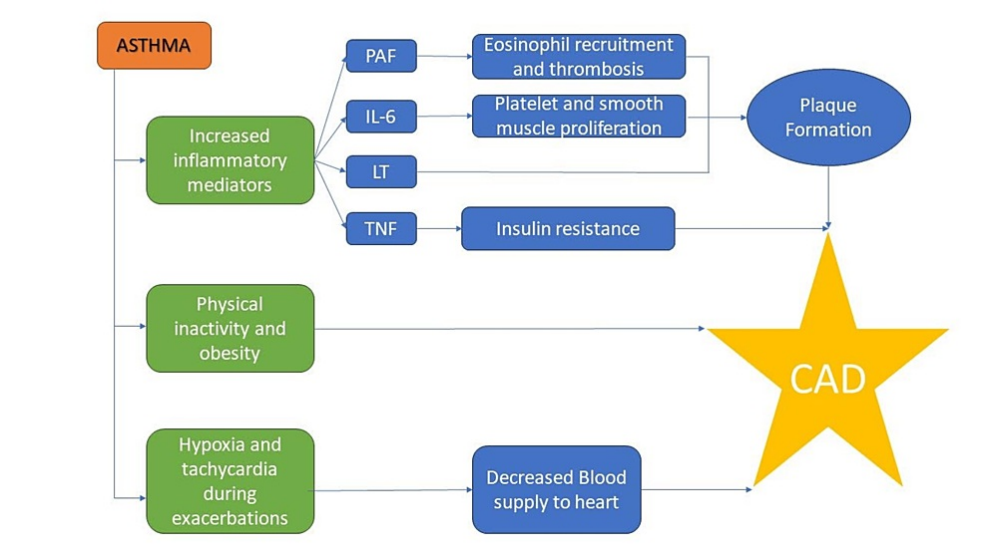
Components of asthma contributing to the pathophysiology of CAD CAD: coronary artery disease, PAF: platelet-activating factor, IL: interleukin, LT: leukotriene, TNF: tumor necrosis factor Image credits: Vasudha S. Garg and Priyansh Patel

The risk of MI may also be increased by platelet-activating factor (PAF) or particular eicosanoid mediators (such as cysteinyl LTs) released in asthma [18]. PAF has been viewed as a significant inflammatory mediator responsible for airway hyperresponsiveness and airway inflammation because it causes eosinophil mobilization into the lung in asthma and imbalanced inflammation and thrombosis in CAD [18]. A connection between atherosclerosis and asthma has recently attracted attention due to the finding that variations in the genes for 5-lipoxygenase and 5-lipoxygenase-activating protein, two essential genes in the control of LT production, are anticipated for an increased likelihood for atherosclerosis and its main clinical repercussions, ischemic stroke, and MI [19]. Additionally, there is growing recognition that the mast cell, which is the biological signature of asthma, is a frequent component of the damaged arterial wall and an active participant in atherogenesis [19]. The release of pro-inflammatory cytokines by activated monocytes, macrophages, and vascular cells is modulated by estrogen levels, which rise during puberty. LTs produced by mast cells are similarly controlled. This might explain the increased incidence of asthma-associated CAD in females [13]. It has been stated that potential intrinsic and extrinsic pathways are present for the mechanisms of underlying correlations between asthma and CAD. While inflammation constitutes the intrinsic pathway, acute asthma exacerbations causing hypoxia and tachyarrhythmia may constitute the extrinsic pathway causing CAD symptoms [20]. Apart from chronic inflammatory pathways, it is possible that obesity or physical inactivity brought on by asthma symptoms could enhance the risk of MI [18]. Obesity, depression, anxiety, and chronic obstructive pulmonary disease (COPD) are comorbidities that are frequently present in people with asthma and are hypothesized to be the risk factors for MI [3]. Figure 1 summarizes the roles of various components of adult asthma in contributing to the development of CAD.

While much literature is present explaining the pathophysiology of traditional atherogenic CAD, not much study has been found to explain the cause of increased CVsA incidence in asthmatic patients [21]. According to a study, impaired endothelial function and hypercontraction of smooth muscle cells of the vessels are the most likely processes driving the pathogenesis of CVsA. Both pathways have a connection to inflammation, which can be found in obstructive lung illness and CAD [21]. Both bronchospasm and coronary vasospasm are brought on by histamine and allergic hypersensitivity reactions [21]. On top of that, it has been reported that attacks of bronchial asthma, or CVsA, often occur in the early morning due to increased parasympathetic nerve activity and acetylcholine (ACh), a parasympathetic neurotransmitter [22]. A prospective study showed that in CVsA patients with airway hyperresponsiveness, the cumulative dosage at the moment at which respiratory conductance begins to drop is substantially linked with the least dose of ACh that caused more than 90% stenosis in the coronary artery. This raised the possibility that people with CVsA had systemic cholinergic hypersensitivity of both nonvascular and coronary artery smooth muscles [22].

Factors affecting CAD development in adult asthmatics

Not all adult asthmatics are at higher risk of developing CAD. Studies have shown that between asthma control and MI risk, there is a significant dose-response relationship, with the risk being highest in individuals with uncontrolled asthma compared to adults with controlled asthma [23]. This implies that having active asthma and having trouble controlling it are linked to an elevated risk of MI [23]. It has been demonstrated that impairment of ventilatory function, particularly rapid decline, is a distinct risk factor for lethal MI and cardiovascular death [15]. A newly acknowledged systemic inflammatory condition called asthma-COPD overlap syndrome (ACOS) shares the symptoms of both asthma and COPD. In a study, it was pointed out that compared to healthy controls, patients with only asthma and only COPD, patients with ACOS had greater levels of inflammatory markers in their sputum and serum. This explained the increased incidence of CAD in ACOS patients compared to the other groups [24]. Apart from the status of asthma and its severity, the medications used in asthma may also have some impact on CAD development. It has been mentioned that the use of beta-2-agonists raises the risk of adverse cardiovascular events in adult asthmatics [25]. When compared to a placebo, the start of treatment raises the heart rate and lowers potassium levels. These processes, along with other outcomes of beta-adrenergic stimulation, may trigger CAD, ischemia, and sudden cardiac death [25]. Furthermore, chronic oral glucocorticoid therapy in severe asthmatics has been linked to weight gain, visceral obesity, insulin resistance, and lipid abnormalities, all of which are significant risk factors for CAD [15]. On the other hand, several studies have demonstrated that the use of asthma drugs does not increase the risk of MI in people with active asthma. It has been contended that, if incorrect, assigning excessive cardiovascular risk to asthma medications such as short-acting bronchodilators could result in the undertreatment of bronchospasm in individuals who are going through an asthma exacerbation [18].

Limitations

The study is subject to some limitations, such as a lack of high-level evidence such as systematic reviews, randomized controlled trials, and meta-analyses. A small number of available clinical trials served as the basis for all of the research. All the research revealed that sample sizes and measurements varied. The variables and secondary outcomes used in all the research under consideration varied. The fundamental causes of these disparities between studies regarding the role of gender in asthmatics with CAD are unclear, and future research from nations with diverse ethnicities may serve to shed light on this discovery. We may be able to pinpoint treatment targets if we can clarify the clinical importance and mechanism of the pathophysiology of CAD in adult asthma patients. To decrease the cardiovascular mortality of asthmatics, additional research should be done to define the involvement of adult asthma features and their treatments in the development of CAD. Information from papers written in languages other than English was not included in this evaluation because it only contained English-language articles. Studies using animals were also omitted. Only studies done on human subjects were included in the review.

## Conclusions

We found strong evidence to support the role of adult asthma in the development of CAD, attributing the chronic inflammatory nature of both chronic diseases as the primary cause of the pathogenesis. Due to the hormonal effect of estrogen on the inflammatory cells, CAD is more commonly found in the female asthmatic population. More studies should be undertaken to unmask the key regulators of asthma influencing coronary vessel damage so that specific target therapy can prevent catastrophic outcomes in asthmatics. The risk rises in uncontrolled asthmatics or in those with rapid declines in ventilatory function, thus proving the importance of asthma control in avoiding CAD incidences. The role of beta-2-agonists in the progression of CAD is unclear and requires further research.
